# Computational prediction and *in vitro* validation of VEGFR1 as a novel protein target for 2,3,7,8-tetrachlorodibenzo-p-dioxin

**DOI:** 10.1038/s41598-019-43232-4

**Published:** 2019-05-02

**Authors:** Kumaraswamy Naidu Chitrala, Xiaoming Yang, Brandon Busbee, Narendra P. Singh, Laura Bonati, Yongna Xing, Prakash Nagarkatti, Mitzi Nagarkatti

**Affiliations:** 1 Department of Pathology, Microbiology and Immunology, University of South Carolina School of Medicine, Columbia, SC 29208 USA; 2 Department of Earth and Environmental Sciences, University of Milano-Bicocca, Milan, Italy; 3 McArdle Laboratory for Cancer Research, University of Wisconsin-Madison, Madison, WI USA

**Keywords:** Computational biology and bioinformatics, Computational biology and bioinformatics, Immunology, Immunology

## Abstract

The toxic manifestations of 2,3,7,8-tetrachlorodibenzo-p-dioxin (TCDD), an environmental contaminant, primarily depend on its ability to activate aryl hydrocarbon receptor (AhR), which is a ligand-dependent transcription factor belonging to the superfamily of basic-helix-loop-helix DNA-binding proteins. In the present study, we aimed to identify novel protein receptor targets for TCDD using computational and *in vitro* validation experiments. Interestingly, results from computational methods predicted that Vascular Endothelial Growth Factor Receptor 1 (VEGFR1) could be one of the potential targets for TCDD in both mouse and humans. Results from molecular docking studies showed that human VEGFR1 (hVEGFR1) has less affinity towards TCDD compared to the mouse VEGFR1 (mVEGFR1). *In vitro* validation results showed that TCDD can bind and phosphorylate hVEGFR1. Further, results from molecular dynamic simulation studies showed that hVEGFR1 interaction with TCDD is stable throughout the simulation time. Overall, the present study has identified VEGFR1 as a novel target for TCDD, which provides the basis for further elucidating the role of TCDD in angiogenesis.

## Introduction

2,3,7,8-tetrachlorodibenzo-p-dioxin (TCDD) represents the prototypical ligand for aromatic hydrocarbon environmental contaminants which elicit a wide range of toxicity through activation of the aryl hydrocarbon receptor (AhR)^1^. AhR is a basic helix-loop-helix/PAS transcription factor localized in the cytoplasm. Because AhR is expressed in a variety of tissues, it mediates species- and tissue-dependent toxicities, including chloracne, wasting, teratogenicity, immunotoxicity, liver tumor promotion and carcinogenicity^2–6^. More recent studies have shown that AhR activation can not only cause toxicity but also regulate the immune response, specifically the regulation of T cell differentiation^7–9^.

Also, in addition to TCDD, other innocuous ligands for AhR have also been identified such as dietary indoles, indole-3-Carbinol (IC3) that are found in high concentrations in cruciferous vegetables^10–13^. Natural flavonoids present in fruits and vegetables, including apigenin, quercetin, and resveratrol are also known to act as AhR ligands^10,13–15^. There are also known endogenous ligands of AhR that are derived from tryptophan metabolism^16^. The precise mechanisms through which AhR ligands mediate such a wide range of effects following activation of AhR, remain unclear.

The interactions between TCDD and AhR has been very well characterized as follows: i) binding of TCDD or other ligands to AhR leads to dissociation of AhR from the complex of heat shock proteins 90, p23 or heat shock protein 23 and hepatitis B X-associated protein ii) migration of the complex formed by AhR with the TCDD or other ligands to the nucleus iv) in nucleus, AhR builds up a heterodimer with the intranuclear AhR nuclear translocator (ARNT), thereby promoting the transcription of xenobiotic response elements (XRE) for metabolizing TCDD or AhR ligands to render them more hydrophilic facilitating their elimination^17–21^.

Identification of novel protein targets and their validation is one of the key steps in the discovery and development of novel pathways in several diseases. In general, identification of novel potential targets for a chemical compound involves extensive proteomic approach involving comparison of the protein expression profiles in a given cell/ tissue in the presence/absence of the given chemical compound^22^. There are certain unavoidable shortcomings in predicting the novel targets for new chemicals or ligands due to time-consuming and expensive nature of the experimental methods to predict. Several novel computational methods and algorithms have emerged in recent years to predict possible biological targets for ligands^22–27^. A previous study reported diverse proteins such as metallopeptidases 8 and 3, oxidosqualene cyclase and myeloperoxidase as theoretical targets for TCDD using Bioinformatics approach^28^.

Because TCDD has been shown to be involved in the regulation of diverse biological, biochemical and immunological pathways, in the current study, we investigated additional protein targets for TCDD using computational approach followed by validation using *in vitro* blocking and phosphorylation experiments. We identified VEGFR1 as a novel target for TCDD, which provides the basis for further investigating the role of TCDD in angiogenesis.

## Results

### Identification of protein targets for TCDD

**Figure 1:**
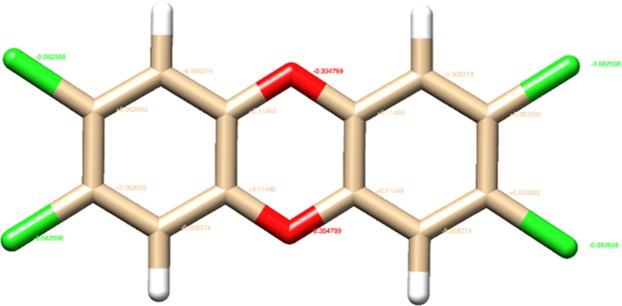
TCDD with Gasteiger partial charges added using Chimera version 1.11^18^.

To study potential receptors for TCDD other than the well characterized AhR, we first downloaded TCDD structure which was optimized by assigning Gasteiger partial charges with AMBER ff99SB force field and converted it into mol2 format using Chimera 1.11^19^ (Fig. 1) and initially subjected to reverse pharmacophore analysis using PharmMapper^29^. Results from PharmMapper along with their respective normalized fit score, are provided in the Supplementary Table S1a,b. PharmMapper derives the pharmacophore models from the structures within PDB^18^. Moreover, we submitted TCDD to SwissTargetPrediction server which combines both 2D and 3D similarity measures with known ligands for knowledge-based prediction of potential targets^30^. Results from molecular target prediction by SwissTargetPrediction tool provided several possible interacting targets for TCDD in *Mus musculus* and *Homosapiens*. These include VEGFR1 and AhR, as well as several other targets as detailed in Supplementary Table 2a,b. Among these predicted targets, VEGFR1 and AhR showed a high probability of interaction in *Mus musculus* whereas in *Homosapiens* Vascular endothelial growth factor receptor 3, Vascular endothelial growth factor receptor 2 along with VEGFR1 and AhR showed a high probability of interaction. The other receptors showed low (range: 0–0.08) probabilities of interaction with targets based on ChEMBL database. Results from SwissTargetPrediction by homology showed that TCDD was predicted to interact with enzymes (67%), Protease (13%), kinase (7%), transcription factor (7%) and cytosolic (7%) in *Mus musculus* (Supplementary Fig. S1A). In contrast, TCDD was predicted to interact with enzymes (53%) and kinase (20%) in *Homosapiens* (Supplementary Fig. S1B). Together, these results indicated that VEGFR1 could be a potential target for TCDD in both *Mus musculus* and *Homosapiens* based on ChEMBL database.

### Homology modeling, structure refinement and validation

**Figure 2:**
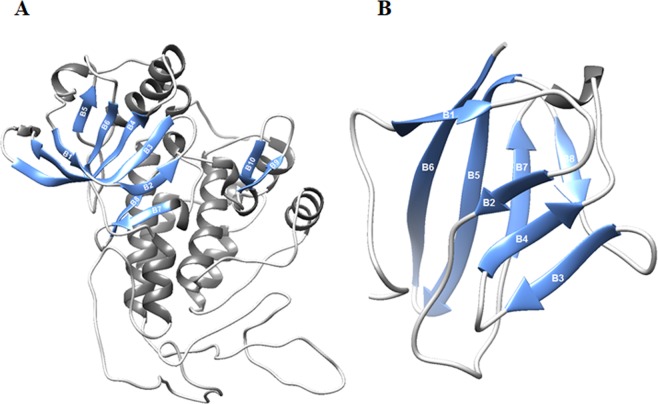
Three-dimensional structure of VEGFR1. (**A**) Represent the three-dimensional structure of mouse VEGFR1 generated using homology modeling (**B**) Represent the X-ray crystal structure of human VEGFR1 domain 2 from PDB (PDB ID: 1RV6, chain X). Helices were represented in dim grey; Beta sheets were represented in corn flower blue; Coils were represented in grey color; VEGFR1 protein is represented in ribbon.

To date, there are no experimental structural data available for the mVEGFR1. To elucidate the structural insight of mVEGFR1, we predicted the three-dimensional structure using homology modeling (Fig. 2A) based on the template structure of hVEGFR1 in complex with a ligand (PDB ID: 3HNG). For hVEGFR1, the X-ray crystal structure of placental growth factor in complex with domain 2 of VEGFR1^31^ downloaded from protein databank (PDB ID: 1RV6) was considered for our study (Fig. 2B). The generated three-dimensional model for mVEGFR1 was validated using the Ramachandran plot. Results from Ramachandran plot analysis showed 97.8% of the residues (352 amino acids) in favoured region, 1.9% of the residues (7 amino acids) in allowed region and 0.3% of the residues (1 amino acid) in outlier region (Fig. S2A). One of the parameter to represent and measure the overall quality and deviation of the total energy of the protein structure is Z-score which is dependent upon the length of protein^28^. Results from PROSA web analysis showed the Z-score of the mVEGFR1 is displayed in the plot with a dark black point (Fig. S2B). The Z-score value of the generated mVEGFR1 model is −6.37, which is within the acceptable range −10 to 10 and is located within the space of proteins related to the NMR and X-ray (Fig. S2B). This is very close to the Z score value −7.44 of the template structure (3HNG) indicating that the generated model is reliable and close to the experimentally elucidated structure (Fig. S2B). Another stereochemical test to assess the quality of a modelled structure is ERRAT, which analyses the dimension of structural error for each residue and statistics of non-bonded interactions between different atoms in a 3D Structure model. Results from ERRAT plot analysis showed an overall quality factor of 80.240 indicating that the generated model is stable and reliable (Fig. S2C). Overall, the results from structure validation parameters demonstrated that the generated 3D model for mVEGFR1 is reliable for our study (Fig. S2A–C).

### Identification of binding pocket amino acid residues for mouse and human VEGFR1

Residues in the crystallographic structures of proteins containing existing or predicted binding pockets play a key role in predicting the binding pocket for the new targets receptors and can be used as an input for such predictions^32^. It is known that toxicities induced by TCDD are mediated through AhR, a ligand-activated transcription factor belonging to the basic helix-loop-helix-PAS family^30^. Among the different domains of AhR, ligand binding domain plays a key role in binding to the TCDD^33,34^. Therefore, initially we aligned the sequences of mVEGFR1 and hVEGFR1 with that of the AhR ligand binding domain to predict the conserved amino acids and calculated the total number of atoms belonging to these amino acids, TC_a_ (Supplementary Table S3). To identify the pocket sites and to elucidate the atoms and amino acids lining each pocket, we used three binding pocket prediction servers: CastP, a web server^35^; Active site, an online server which calculates different cavity sites of a protein and analyses the xyz coordinates on the cavity; and FTsite, a web based server that calculates the binding site residues using solvent mapping algorithm^36,37^. Scoring results (Supplementary Data 1), of CastP and Active site servers were provided in the Supplementary Tables S4 and S5. Results from FTsite were shown in the Supplementary Fig. S3A,B. Molecular surface representation of pocket residues predicted using scoring function is provided in the Supplementary Fig. 4C,D.

### Molecular docking studies

**Figure 3:**
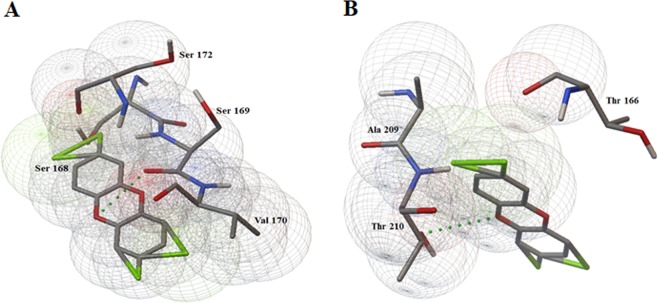
Docking poses of VEGFR1 interaction with the TCDD. (**A**) Represents the interaction of TCDD with mVEGFR1. TCDD showed interacting with the side chains of Ser 169 in mVEGFR1 by forming hydrogen bonds (**B**) Represents the interaction of TCDD with hVEGFR1. TCDD showed interacting with the side chains of Thr 210 in hVEGFR1 by forming hydrogen bonds. AhR ligand binding domain residues were shown in sticks with their contact surfaces being shown in ball wire frame representation.

We performed molecular docking studies to test whether TCDD shows affinity and inhibitory activity towards the mouse and human VEGFR1 in the pockets predicted using scoring function. Results from molecular docking studies showed that TCDD interacts with mVEGFR1 with a binding energy of −7.41 kcal/mol and inhibition constant Ki of 3.68 µM and it interacts with the hVEGFR1 with a binding energy of −6.59 kcal/mol and estimated inhibition constant Ki of 14.8 µM. Results showed that TCDD forms hydrogen bonding interactions with the side chains of Ser 169 residue in mVEGFR1 and with the side chains of Thr 210 residue in hVEGFR1 (Fig. 3). These results indicated that TCDD has stronger binding affinity towards mVEGFR1 compared to hVEGFR1. Further, crystal structure of mVEGFR1 is not available and only a part of mVEGFR1 as recombinant mouse soluble VEGFR-1_D7_/Fc chimera is available^38,39^. Therefore, we conducted experimental studies followed by molecular dynamic simulations on hVEGFR1.

### *In vitro* validation of TCDD binding, blocking and phosphorylation with hVEGFR1

**Figure 4:**
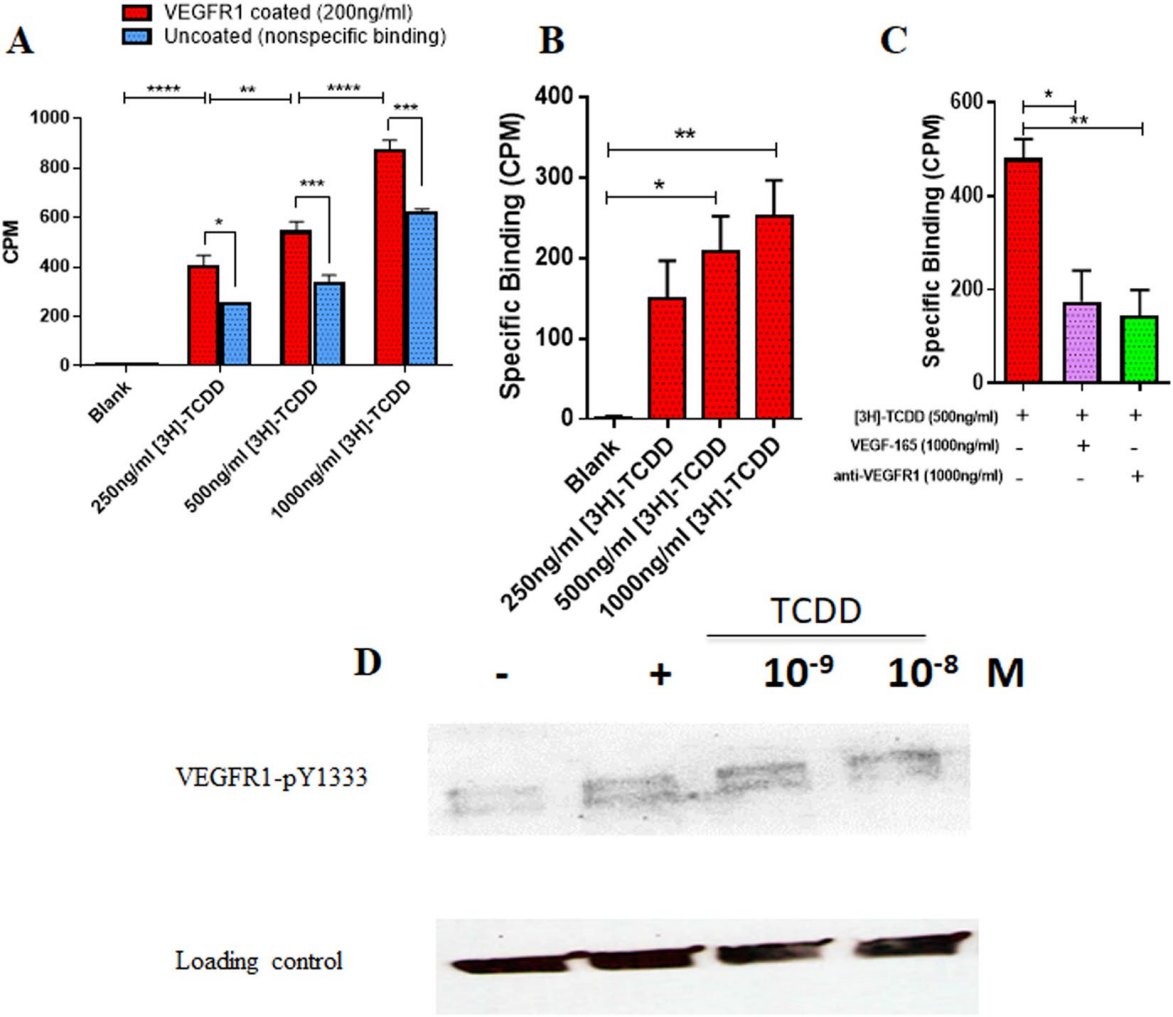
*In vitro* blocking, binding and Phosphorylation of hVEGFR1. (**A**) Represents control uncoated (red bars) and VEGFR1-coated wells (blue bars) incubated with varying doses (250 ng/ml–1000 ng/ml) of [3 H]-TCDD to detect potential binding (**B**) Represents specific binding, readings (in CPM) from uncoated controls were subtracted from those coated with VEGFR1 (**C**) Represents specific binding of TCDD to VEGFR1. VEGFR1-coated wells were incubated with vehicle only (DMSO), VEGF-165 (1000 ng/ml), or anti-VEGFR1 (1000 ng/ml) prior to incubation with [3 H]-TCDD. Results are representative of three independent experiments. Statistical significance (P-value: * < 0.05, ** < 0.01, *** < 0.005, **** < 0.001) was determined with either one-way or two-way ANOVA analysis followed by Tukey’s post hoc multiple comparisons test (**D**) HUVEC cells were starved for 24 hr, then 10% serum was added to the medium as a positive control. TCDD with the indicated concentration was added to the other samples. Vehicle treated sample was used as a negative control. Samples were collected after 10 min of treatment, the phosphorylated VEGFR1 was determined by Western blot.

To experimentally validate our molecular docking studies on interactions between TCDD with hVEGFR1, we conducted modified ELISA-based assay approaches *in vitro* using [3 H]-TCDD and recombinant human VEGFR1. Results showed that [3 H]-TCDD levels significantly increased in a dose-dependent manner in VEGFR1-coated wells (Fig. 4A). The detection of [3 H]-TCDD was significantly increased at all three doses (250, 500, 1000 ng/ml) compared to respective non-coated well counterparts, indicating the specific binding of TCDD with hVEGFR1 (Fig. 4B). To check whether this specific binding of TCDD to hVEGFR1 could be blocked using antibody or another ligand, such as VEGF-165, specific for this receptor, we performed blocking studies with VEGF-165. Results showed that the wells, pre-incubated with either VEGF-165 or anti-VEGFR1 (1000 ng/ml) showed decreased levels of [3 H]-TCDD compared to those coated with only TCDD (Fig. 4C). These results indicated that TCDD specifically binds to hVEGFR1 and that this binding can be blocked with either competing ligands (VEGF-165) or antibodies for the receptor. Further, results from the phosphorylation of VEGFR1 *in vitro* in HUVEC cells, showed that TCDD can induce VEGFR1 phosphorylation (Fig. 4D), suggesting that the receptor may indeed be activated by TCDD. Overall, these results demonstrated that TCDD can specifically bind, block and phosphorylate hVEGFR1.

### MDS of hVEGFR1 and hVEGFR1-TCDD complex

**Figure 5:**
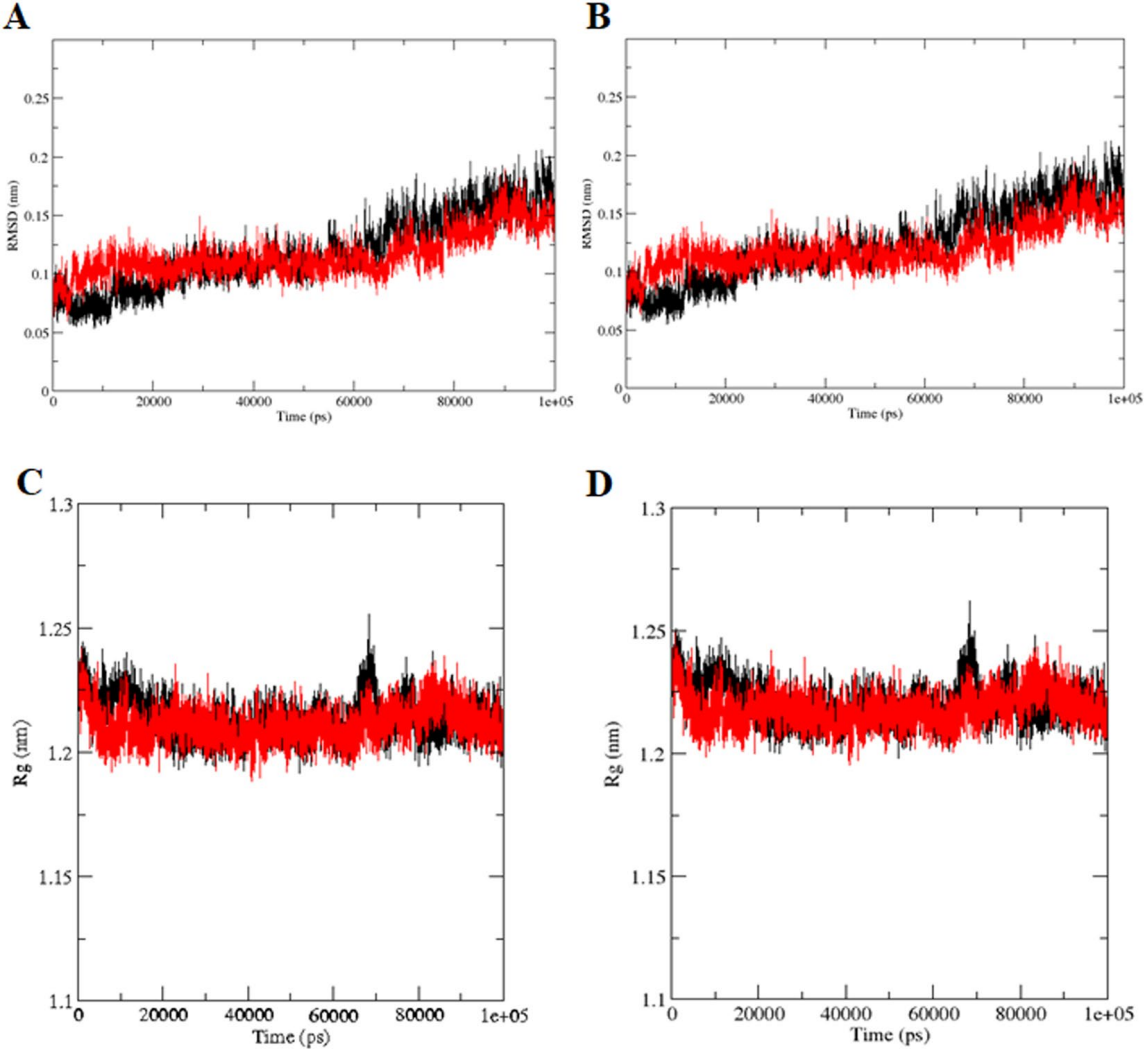
RMSD and Rg plots of hVEGFR1 and hVEGFR1-TCDD complexes. (**A**) Represents backbone RMSD (**B**) Represents Cα RMSD (**C**) Represents backbone Rg (**D**) Represents Cα Rg. hVEGFR1 is shown in black color, hVEGFR1-TCDD complex is shown in red color.

Understanding the binding of a ligand typically to a substrate or a regulator is a dynamic process and is key in understanding the function itself^32^. MDS has been used successfully in elucidating ligand-induced perturbations in several studies reported previously^35–39^. In the present study, to analyse the conformational changes between hVEGFR1 and hVEGFR1-TCDD complexes, we performed MDS. To determine the stability of interactions and mechanistic aspects of hVEGFR1-TCDD interaction, we performed RMSD and Rg calculations. Figure 5A,B demonstrated the RMSD of the backbone and C-alpha residues in hVEGFR1 and hVEGFR1-TCDD complex. The magnitude of fluctuations indicated that both hVEGFR1 and hVEGFR1-TCDD complex backbone and C-alpha systems attained stable equilibration conformations during the first 20–60 ns and showed an increase during the last 20 ns of the simulation (Fig. 5A). Results showed that there is not much change in back bone (0.11 ± 0.0 nm) and C-alpha (0.12 ± 0.0 nm) average RMSD for hVEGFR1-TCDD complex compared to hVEGFR1 (0.11 ± 0.0 nm, 0.12 ± 0.0 nm). These results demonstrated that TCDD doesn’t influence the stability of hVEGFR1 system upon binding. To analyse the compactness or spatial spread of protein mass^36^ for each protein system upon binding to TCDD during the simulations, we calculated the Rg of backbone and C-alpha alpha atom for hVEGFR1 and hVEGFR1-TCDD complex systems. Figure 5C,D shows the Rg of the backbone and C-alpha residues in hVEGFR1 and hVEGFR1-TCDD complex. Results showed that there is no significant difference in the back bone (1.21 nm) and C-alpha (1.21 nm) average Rg for hVEGFR1-TCDD complex compared to hVEGFR1 (1.21 nm, 1.22 nm). These results demonstrated that TCDD binding doesn’t influence on the stability and compactness of hVEGFR1 system. Overall, these results showed that the binding of TCDD did not affect the overall conformational diversity of the hVEGFR1 protein system significantly.

**Figure 6:**
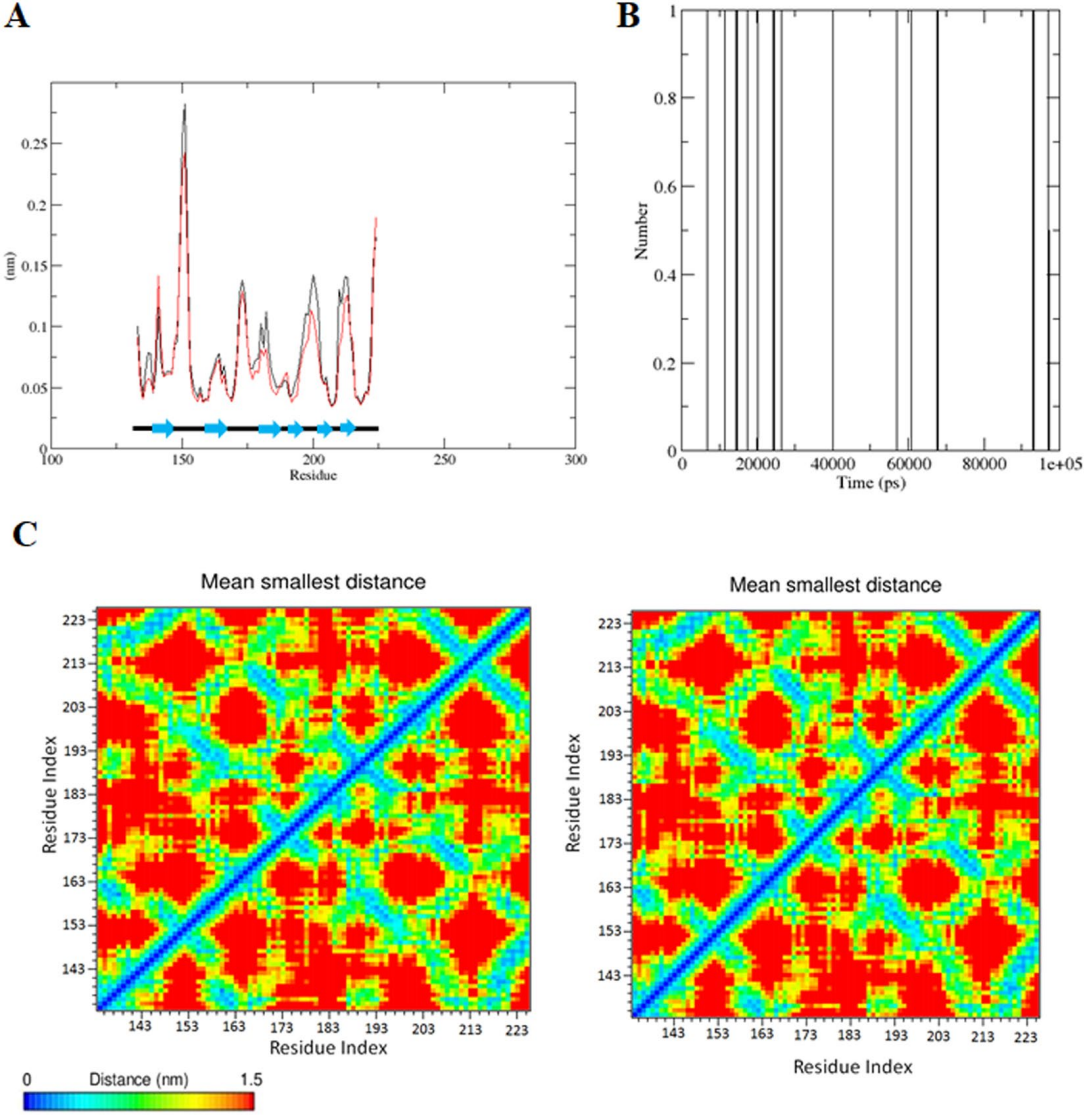
RMSF, hydrogen bonding interactions and mean smallest distance plots of hVEGFR1 and hVEGFR1-TCDD complexes. (**A**) Backbone RMSF per residue for the structures during molecular dynamics calculations (**B**) Represents the nature of the hydrogen-bond interactions during the MDS (**C**) Mean smallest distances calculated between residues observed during MD simulation in hVEGFR1 (left) and hVEGFR1-TCDD complex (right). Shorter distances are shown with blue, green and yellow colors, and longer distances are shown in red.

To predict the residue regions in hVEGFR1 exhibiting higher flexibility, we calculated the RMSF per residue parameter. Figure 6A shows the RMSF of the backbone residues in hVEGFR1 and hVEGFR1-TCDD complex. Results showed a higher fluctuation in the loop regions (residues around 150, 175) and β-sheet regions (residues around 200, 216) with largest fluctuations occurring at the loop regions (Fig. 6A). These results again confirmed that the binding of TCDD did not significantly affect the hVEGFR1 protein system overall conformational diversity. Our molecular docking results showed a hydrogen bond between TCDD and Thr 210 residue of the hVEGFR1. Using the g_hbond tool of GROMACS, we determined the number of hydrogen bonds and their occupancy in the hVEGFR1-TCDD complex during the MDS. Figure 6B shows the hydrogen bonds in the hVEGFR1-TCDD complex during the MDS. Results showed that the hydrogen bond formed between Thr 210 and hVEGFR1 was stable during the first 20 ns but fluctuated substantially during the later last 20 ns of MD simulations (Fig. 6B). Overall, these data showed that the hydrogen bonds at the docking level (Fig. 3B) were maintained during the MD simulations. To characterize the global effect of TCDD on the hVEGFR1 dynamics, we calculated the fluctuations of pairwise amino acid distances on all possible residue pairs in both hVEGFR1 and hVEGFR1-TCDD complex. Figure 6C shows mean contact distances between residues observed during MD simulation in hVEGFR1 and hVEGFR1-TCDD complex. Results showed that the patterns of flexibility/rigidity in the hVEGFR1-TCDD complex are highly similar to the ones in the hVEGFR1 only (Fig. 6C). Results showed that there is small difference in the average mean distance between the residues in the hVEGFR1-TCDD (1046.38) complex compared to the hVEGFR1 (1047.92), thereby demonstrating that TCDD binding may be mechanistically relevant and may regulate the functionally oriented aspects of hVEGFR1 conformational dynamics.

## Discussion

Several chemical moieties bind to more than one target, which can be distinct in terms of their both sequence and functionality^18^ and mapping the actual protein targets for small molecules is therefore a key in better understanding their mechanism of action. Some chemicals or molecules may bind to more than one target and predicting such secondary targets will be useful in exploiting their activity on other proteins than those they were initially well known to bind to^1^. In the present study, we used both *in silico* and *in vitro* approaches to predict and confirm new protein targets for TCDD.

Initially we used PharmMapper, a web based server that uses Pharmacophore approach for predicting novel protein targets for the chemicals or small molecules by integrating information in TargetBank, BindingDB, DrugBank and potential drug target database, including over 7000 receptor-based pharmacophore models^29^. PharmMapper has been successful in previous studies such as for predicting the anti-inflammatory effects of Shufengjiedu Capsule^10^, antitumor evaluation of some new 1,2,4-triazines^27^, inhibition of lipopolysaccharides-induced nucleotide-binding domain leucine-rich repeats family pyrin domain-containing 3 (NLRP3) inflammasome activation by hydroxysafflor yellow A^12^, identification of a potential target for Capsaicin^13^, identification of anti-cancer targets for Punica granatum^14^, and several similar targets^15,16,40^. Results from our analysis using PharmMapper showed no identical protein targets for TCDD in both human and mouse (Supplementary Table S1a,b). To predict common protein targets in both human and mouse, we submitted TCDD to SwissTargetPrediction, a web server that accurately predicts the targets of bioactive molecules by combing the 2D and 3D similarity with known ligands and maps prediction by homology within and between different species^30^. SwissTargetPrediction has been successful in previous studies such as for predicting anti-cancer and anti-leishmanial targets for carboxylate derivatives of tributyltin (IV) complexes^41^ and predicting new targets for bioactive small molecules^18^. Results from SwissTargetPrediction showed VEGFR1 as identical protein target for TCDD other than AhR predicted in both human and mouse (Supplementary Table S2a,b).

For mVEGFR1, we generated three-dimensional model using homology model (Fig. 2A) whereas for hVEGFR1 we downloaded the X-ray crystal structure from PDB (Fig. 2B). To analyse the mechanism of interaction between the mouse and human VEGFR1 with TCDD, we performed molecular docking calculations. Molecular docking has been successful previously in several studies including elucidating the binding mechanism of looped host defence peptide CLP-19 to microtubules^42^, novel inhibitors of anthrax edema factor^43^, exploring the molecular mechanism of pathogenesis caused by *Pseudomonas aeruginosa*^44^ and several other studies^7,45,46^. Results from our molecular docking experiments showed that mVEGFR1 has high affinity towards TCDD compared to hVEGFR1 (Fig. 3). Further, results from the molecular docking studies were validated *in vitro* using the binding assays and phosphorylation of TCDD with hVEGFR1. Results showed that TCDD can specifically bind to hVEGFR1 (Fig. 4A–C) and phosphorylate hVEGFR1 (Fig. 4D).

To analyse and explore the underlying structural features and mode of action of TCDD -hVEGFR1 interactions, we performed molecular dynamics simulations (MDS). In principle, MDS provides the time evolution of atomistic models of proteins^8,47,48^. MDS has been successful in several studies including analysis of interactions of a novel benzenediamine derivate FC-99 interaction with its target in experimental sepsis^9^ and in studying the interferon and innate immunity resistance in Middle East Respiratory Syndrome Coronavirus Non-Structural Protein 3^49^. To study the stability of interaction, we calculated the RMSD, Rg, RMSF, hydrogen bonding and mean residue distance parameters for hVEGFR1 and hVEGFR1-TCDD complex. Our results showed that the hVEGFR1-TCDD complex interaction was stable throughout the simulation (Figs 5 and 6). Overall, results from MDS showed that TCDD interaction with hVEGFR1 was stable and may functionally regulate the conformational dynamics of hVEGFR1.

Vascular endothelial growth factor (VEGF) is proangiogenic and is critical for the growth and survival of vascular endothelium. The VEGF family includes VEGF-A, VEGF-B, VEGF-C, VEGF-D, and placenta growth factor (PlGF)^50^. VEGFs bind primarily to three endothelial transmembrane receptors, VEGFR1, VEGFR2, and VEFGR3. VEGFR1/FLT-1 (fms-like tyrosine kinase) belongs to the receptor tyrosine kinases (RTK) subfamily^50^. VEGFR1 plays a crucial role in normal development and angiogenesis. Thus, targeted deletion of VEGFR-1, results in early embryonic lethality due to abnormal blood vessel growth^42^. However, it is noteworthy that VEGFR1 may play both negative and positive roles in angiogenesis^43^. Because VEGFR1 is expressed on both endothelial and nonendothelial cells such as immune cells and tumor cells, it may be associated with additional functions such as inflammation and tumorigenesis^44,45^. Recent studies also found that pharmacological inhibition and genetic deletion of endothelial VEGFR1 enhanced adipose angiogenesis and in a diet-induced obesity model, endothelial-VEGFR1 deficiency exhibited a potent anti-obesity effect^46^.

In this context, our results demonstrating that TCDD binds to VEGFR1, is highly significant and suggests that this pathway may play a role in the regulation of angiogenesis, inflammation and cancer. It is well established that TCDD is toxic to vascular development especially during embryonic stage, which has been seen in several species tested^40^. However, there are very few published reports that link TCDD or its receptor, AhR, to VEGF family. For example, AhR agonists or TCDD were shown to increase VEGF secretion in bronchial epithelial cells^41^ and in the ocular tissues^51^, consistent with our previous studies demonstrating that treatment of mice with TCDD enhanced the expression of several genes involved in angiogenesis, specifically VEGF^52^. In contrast, TCDD-mediated coronary vascularization was found to be associated with reduced VEGF-A secretion. A previous report showed that TCDD differentially suppresses the angiogenic responses in human placental vein and artery endothelial cells^50^. Furthermore, compounds such as [3-(3,5-dimethyl-1H-pyrrol-2-ylmethylene)-1,3-dihydro-indole-2-one] (SU5416) were shown to have activity as a VEGFR-2 inhibitor and an AhR agonist^34^. Because VEGFR1 acts as a decoy receptor, with potentially numerous functions, our studies on interaction between TCDD and VEGFR1 opens several new avenues of research.

## Conclusion

TCDD is an environmental contaminant well characterized for its toxicity mediated through activation of AhR. In this study, we make an exciting observation that VEGFR1 could be a possible target for TCDD. This combined with previously published studies that TCDD can disrupt the VEGF pathway and impact angiogenesis suggests that some of its actions on angiogenesis may result from direct binding and activation of VEGFR1. These studies open up new pathways of analysis of TCDD-VEGFR1 interactions in the regulation of angiogenesis, inflammation and tumorigenesis.

## Methods

### Molecular target prediction for TCDD

The three dimensional structure file for TCDD was downloaded from the Pubchem database (PubChem CID: 15625), a database resource to analyze the bioactivity of small molecules^53,54^. Target receptors for TCDD was predicted using PharmMapper^29^, a web based server for identifying the potential targets of a given molecule, and SwissTargetPrediction^30^, a knowledge-based approach to predict new targets of an uncharacterized molecule or secondary targets for a known molecule, computationally. PharmMapper identifies the potential targets for a given molecule using reverse pharmacophore mapping approach where target proteins with highest fit scores between corresponding pharmacophore models and query compounds are predicted as potential targets^33^. SwissTargetPrediction predicts the targets for bioactive molecules based on combination of 2D and 3D similarity measures with known ligands. The predictions can be carried out in five different organisms including *Homo sapiens*, *Mus musculus*, *Rattus norvegicus*, *Bos Taurus* and *Equus caballus*^30^.

### Homology modeling, structure refinement and validation

Three-dimensional structure for mVEGFR1 is not available. Therefore, the amino acid sequence of mouse VEGFR1 (mVEGFR1), was downloaded from the Uniprot database^55^ (UniProtKB entry: P35969) and subjected to homology modeling. Amino acid residues from 797–1158 were used for model generation. To model the mVEGFR1 structure, the crystal structure of the human VEGFR1 in complex with N-(4-Chlorophenyl)-2-((pyridin-4-ylmethyl)amino)benzamide (PDB ID: 3 HNG) was chosen as a template structure which shared a sequence identity of 92% (the sequence identity was calculated by the NCBI blastp-suite (https://blast.ncbi.nlm.nih.gov/Blast.cgi)^56^ with the target sequence. To generate homology models for mVEGFR1, MODELLER 9.14^57^ program was used. The quality of the generated mVEGFR1 homology models were assessed using RAM-PAGE sever^58^ for stereo chemical geometry and ProSA web server^59^ for energy assessment. RMSD between the model and the template was calculated using the CLICK web server (http://mspc.bii.a-star.edu.sg/minhn/click.html)^60^. The amino acid environment of modelled structure was evaluated using ERRAT program plots^26^, which assess the distribution of different types of atoms with respect to one another in the protein model and determines the false statistics of bad non-bonded interactions. The validated model was subjected to energy minimization using NOMADRef server^61^ with conjugate gradient method and chosen for further study. For human VEGFR1 (hVEGFR1), crystal structure of placental growth factor in complex with domain 2 of vascular endothelial growth factor receptor-1(PDB ID: 1RV6)^31^ downloaded from Protein Data Bank (PDB)^22^ was used for further study.

### Prediction of Binding Pockets

To predict the binding site for VEGFR1 with TCDD, we integrated the steps followed by the DrosteP^62^ and Metapocket^63^ algorithms. The following three aspects were used for predicting the binding pockets for VEGFR1 (a) alignment of the protein sequence of AhR ligand binding domain with the sequence of protein targets (b) Prediction of the binding pockets in both the modelled and the crystallographic structures of VEGFR1 by the servers CASTp, Active Site and FTsite (c) choosing a scoring scheme for selecting the most reliable binding pockets.

#### Alignment of the sequences

For each VEGFR1 protein target with known (hVEGFR1) and built three dimensional structure (mVEGFR1), a pair wise sequence alignment was performed with the AhR Ligand binding domain protein sequence using EMBOSS Needle with default settings; a web based sever to create an optimal global alignment of two sequences, using the Needleman-Wunsch algorithm available at (http://www.ebi.ac.uk/Tools/psa/emboss_needle/)^64^. From the alignment, the numbers of conserved and identical amino acids were identified and the total number of atoms belonging to the conserved and identical amino acids, TC_a_ was calculated.

#### Prediction of interior cavities and surface pockets

For each protein target with known and built three dimensional structure, we used CastP^35^, Active site (http://www.scfbio-iitd.res.in/dock/ActiveSite_new.jsp) and FTsite^65^ to identify the pocket sites and to elucidate the atoms and amino acids lining each pocket.

#### Scoring scheme

Because the CastP and Active site programs use different scoring functions and are hard to evaluate the predicted pocket sites, we used the same procedure followed by the Metapocket. We made the ranking scores by calculating a z-score separately for each site in both methods and the top three ranked pocket sites in each method were taken into further consideration. The scoring function and parameters used for predicting the residues lining the binding pocket was provided in the Supplementary Data 1. From the top three pockets predicted from the binding pocket prediction programs, we calculated the observed pocket conservation OP_c_ i.e., the observed number of atoms lining the pocket and belonging to amino acids that are identical or conserved in the alignment. We estimated the expected pocket conservation EP_c_ i.e., total number of atoms belonging to conserved or identical amino acids in the alignment with AhR ligand binding domain (TC_a_) multiplied by the number of atoms lining pocket (P_a_) divided by the total number of atoms of the protein (T_a_) (EP_c_ = TC_a_*P_a_/T_a_). As a descriptor of statistical significance, we used Poisson distribution (λ^OPc^/^EPc^ e^−λ^/(OP_c_/EP_c_)! where λ is the population mean) and Poisson p-value (e^−EPc^ EP_c_^OPc^/OP_c_!) to optimize the amino acids in the pocket. The amino acids with highest OP_c_/EP_c_ and lowest p-value were predicted to be the amino acids in the active pocket (Supplementary Table S3).

### Molecular docking

Molecular docking studies were performed using the Autodock 4.2 software^66^. The input files for the molecular docking were generated using PyRx program^67^. For the docking using empirical free energy function and Lamarckian genetic algorithm calculation, we used the following settings: a maximum number of energy evaluations of 2,500,000, an initial population with randomly placed individuals of 150, a maximum number of generations of 27,000, a mutation rate of 0.02, a crossover rate of 0.8, and an elitism value of 1, Solis and Wets algorithm with a maximum iteration per search for local search of 300. For all the other parameters not mentioned here, we used the default values.

### TCDD binding and blocking assays

In order to test the ability of TCDD to bind VEGFR1, we used tritiated TCDD ([3H]-TCDD) (American Radiolabelled Chemicals Inc., St. Louis, MA) along with a modified ELISA-based assay system as reported elsewhere^21,24^. In these studies, recombinant hVEGFR1 full-length protein (Biolegend, San Diego, CA) that contained the predicted TCDD binding site (Fig. S4) was coated on wells of white 96-well optical plates (Thermo Fisher Scientific, Waltham, MA) at a concentration of 200 ng/ml overnight. For controls, to account for nonspecific binding of [3 H]-TCDD, wells were left uncoated. Once coated, wells were washed 3x with 300 µl of PBS with 0.5% Tween 20. Plates were then blocked for 2 hours with 2% bovine serum albumin (BSA), followed by repeat washing as previously done. Then, wells were incubated with [3 H]-TCDD (250–1000 ng/ml) for one hour followed by appropriate washing using a Perkin Elmer 96 cell Filter mate harvester (Waltham, MA). To detect binding of [3 H]-TCDD, 200 µl of Perkin Elmer Beta plate Scint cocktail was added to each well and counts per minute (CPM) were detected using a Perkin Elmer 1450 LSC & Luminescence Microbeta Trilox counter. To determine specific binding of [3 H]-TCDD, CPM readings of [3 H]-TCDD from non-coated wells (nonspecific binding) were subtracted from CPM readings from respective VEGFR1-coated wells. For blocking assays, the previous steps were repeated until blocking with 2% BSA. After this blocking and subsequent wash step, wells were incubated for 1 hour with either a well-known ligand for VEGFR1, a splice variant of VEGF-A known as VEGF-165 (Biolegend, San Diego, CA)^25^ or anti-VEGFR1 antibody [AP-MAB0702] (Abcam, Cambridge, MA) at a concentration of 1000 ng/ml. After a wash step, [3 H]-TCDD was added to the wells at 500 ng/ml and incubated for 1 hour before detection of CPM as explained above. A schematic of the binding and blocking assays is depicted in the Fig. S4.

### Western blot analysis

To check the phosphorylation of TCDD with hVEGFR1, we performed western blot analysis. HUVEC cells were purchased from ATCC (Manassas, VA) and cultured using endothelial cell growth medium with VEGF (ATCC# PCS-100–041). When cells reached near 80% confluence, they were incubated in DMEM medium without serum or growth factor for 24 hr. Then 10% serum was added to the medium as a positive control to stimulate VEGFR1 phosphorylation. Cells treated with vehicle (DMSO) was used as a negative control. TCDD with the indicated concentration was added to other samples. After 10 min treatment, cells were washed with PBS and lysed with RIPA buffer (Thermo Fisher). The phosphorylated VEGFR1 was determined by Western blot using anti-phospho-VEGFR1 (PTYR1333) antibody (Sigma). Tubulin was used as a loading control.

### Molecular dynamic simulations (MDS) and trajectory analysis

To investigate the mechanism of structural consequences of the VEGFR1 binding to TCDD in *Homosapiens*, we performed molecular dynamics simulations. The obtained complex from molecular docking of TCDD with VEGF1 in *Homosapiens*, was subjected to molecular dynamics using GROMACS version 5.0.4 with the standard OPLS-AA/L all-atom force field^68,69^ and the flexible SPC water model. The topology parameters for TCDD were generated using the ACPYPE software program^70^. The initial VEGF1, VEGF1-TCDD complexes were immersed in a periodic water box of truncated cubic shape (1 nm thickness) and neutralized with the respective counter ions. We used Particle mesh Ewald method for calculating the electrostatic energy^71^. Cut-off distances for the calculation of the Coulomb and van der Waals interaction were 1.0 and 1.0 nm. Energy minimization was done using a steepest decent algorithm of a maximum step size 0.01 nm respectively and with a tolerance of 1000 kJ/mol/nm. The system was subjected to equilibration at a temperature 300 K and a pressure 1 bar. Finally, the full system was subjected to 100 ns MDS and the atom coordinates were recorded every 2 ps during the simulation for later analyses. Comparative structural deviations in protein (VEGFR1) and protein-ligand complex (VEGFR1- TCDD complex of *Homosapiens*) during the simulations were analyzed. We used g_rms, g_rmsf, do_dssp built-in functions of GROMACS package to compute the root mean-square deviation (RMSD), radius of gyration (Rg), root mean-square fluctuation (RMSF) and secondary structure fluctuations. Further, smallest distance between residue pairs were calculated based on the distance matrices generated using the g_mdmat built in function in GROMACS package. The relative graphs obtained from molecular dynamics simulation were plotted using GRACE software (http://plasma-gate.weizmann.ac.il/Grace/).

## Supplementary information

Supplementary information
