# Supplementary material for: Computational prediction and in vitro validation of VEGFR1 as a novel protein target for 2,3,7,8-tetrachlorodibenzo-p-dioxin
Source: Sci Rep. 2019 May 2;9:6810. doi: 10.1038/s41598-019-43232-4 (PMC6497656; doi:10.1038/s41598-019-43232-4)
Supplement: Supplementary file 1 — Supplementary information [file 41598_2019_43232_MOESM1_ESM.pdf]

Computational prediction and *in vitro* validation of VEGFR1 as a novel protein target for 2,3,7,8-tetrachlorodibenzo-p-dioxin

Kumaraswamy Naidu Chitrala[1], Xiaoming Yang[1], Brandon Busbee[1], Narendra P. Singh[1], Laura Bonati[2], Yongna Xing[3], Prakash Nagarkatti[1], and Mitzi Nagarkatti[1,*]

[1]University of South Carolina School of Medicine, Department of Pathology, Microbiology and Immunology, Columbia, SC 29208, USA
[2]University of Milano-Bicocca, Department of Earth and Environmental Sciences, Milan, 20126, Italy
[3]University of Wisconsin-Madison, McArdle Laboratory for Cancer Research, Madison, WI 53705, USA
[*]Mitzi.Nagarkatti@uscmed.sc.edu

A **B**

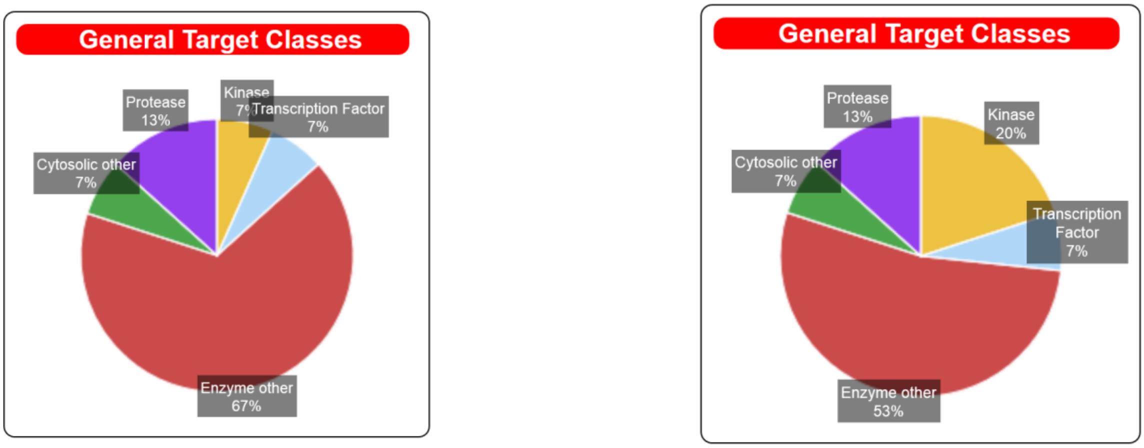

Fig. S1. Molecular targets prediction for TCDD. (A) Pie chart showing the distribution of target classes for mouse. **(B)** Pie chart showing the distribution of target classes for human. Predictions represented in **A, B** are based on the homology.

A

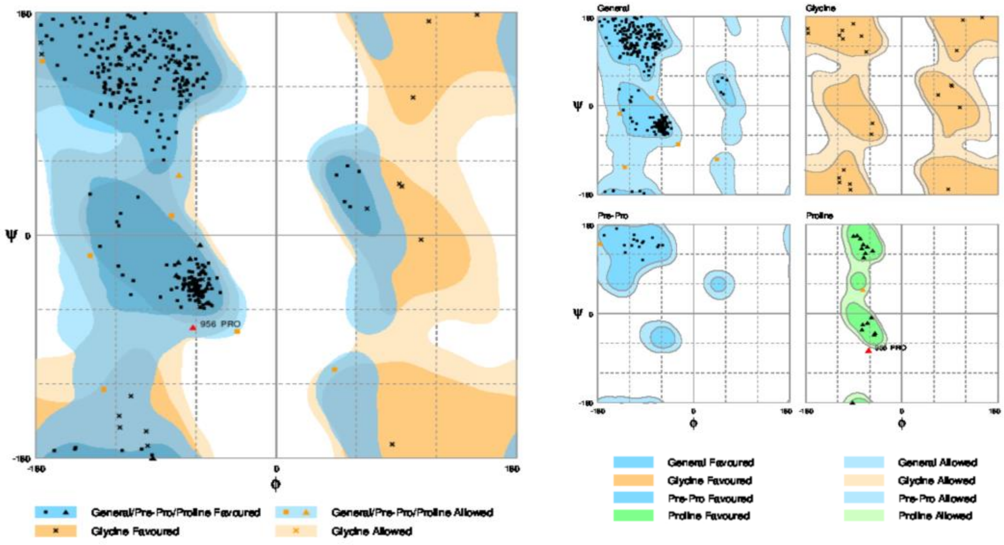
ψ
ϕ
956 PRO
General/Pre-Pro/Proline Favoured
Glycine Favoured
General/Pre-Pro/Proline Allowed
Glycine Allowed
General
Glycine
Pre-Pro
Proline
General Favoured
Glycine Favoured
Pre-Pro Favoured
Proline Favoured
General Allowed
Glycine Allowed
Pre-Pro Allowed
Proline Allowed

B

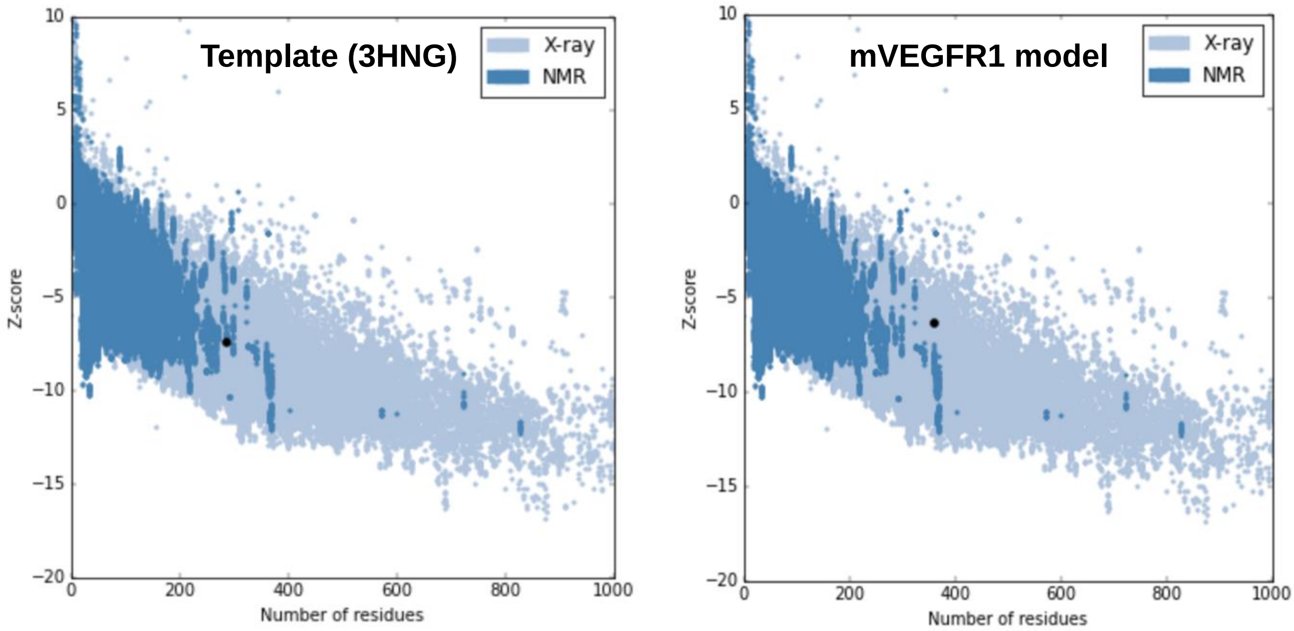
Template (3HNG)
X-ray
NMR
Z-score
Number of residues
mVEGFR1 model
X-ray
NMR
Z-score
Number of residues

C

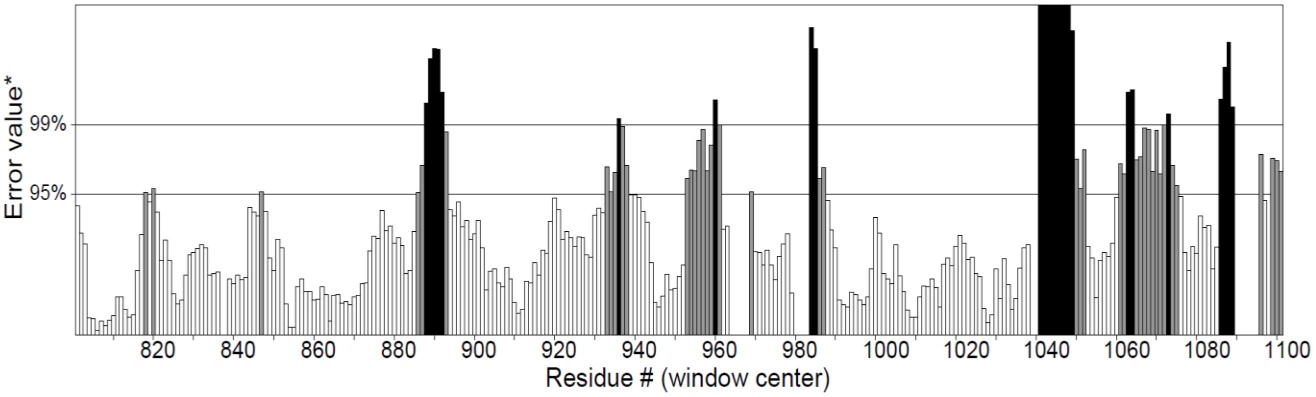
Error value*
99%
95%
Residue # (window center)

Fig. S2. Structure validation of mVEGFR1. (A) Ramachandran plot assessment validation of predicted mVEGFR1 model **(B)** The quality of the generated mVEGFR1 model along with that of template structure used for modeling was evaluated using ProSA-web. The calculated quality (*Z*) scores (closed circles) are displayed in the context of all experimentally determined protein structures available in the Protein Data Bank with each dot representing a distinct structure solved by X-ray crystallography (light blue) or NMR (dark blue). The two black dots represents the Z-score of template and mVEGFR1 model. Results showed a z-score value of -7.44 for the template 3HNG and -6.37 for the built mVEGFR1 homology model **(C)** ERRAT plot for residue-wise analysis of mVEGFR1 homology model with a overall quality factor 80.240. The grey bars in the plot indicate the region of error, the black bars indicate the misfolded area and white bars in the plot indicate the region for folding of protein having less error rate.

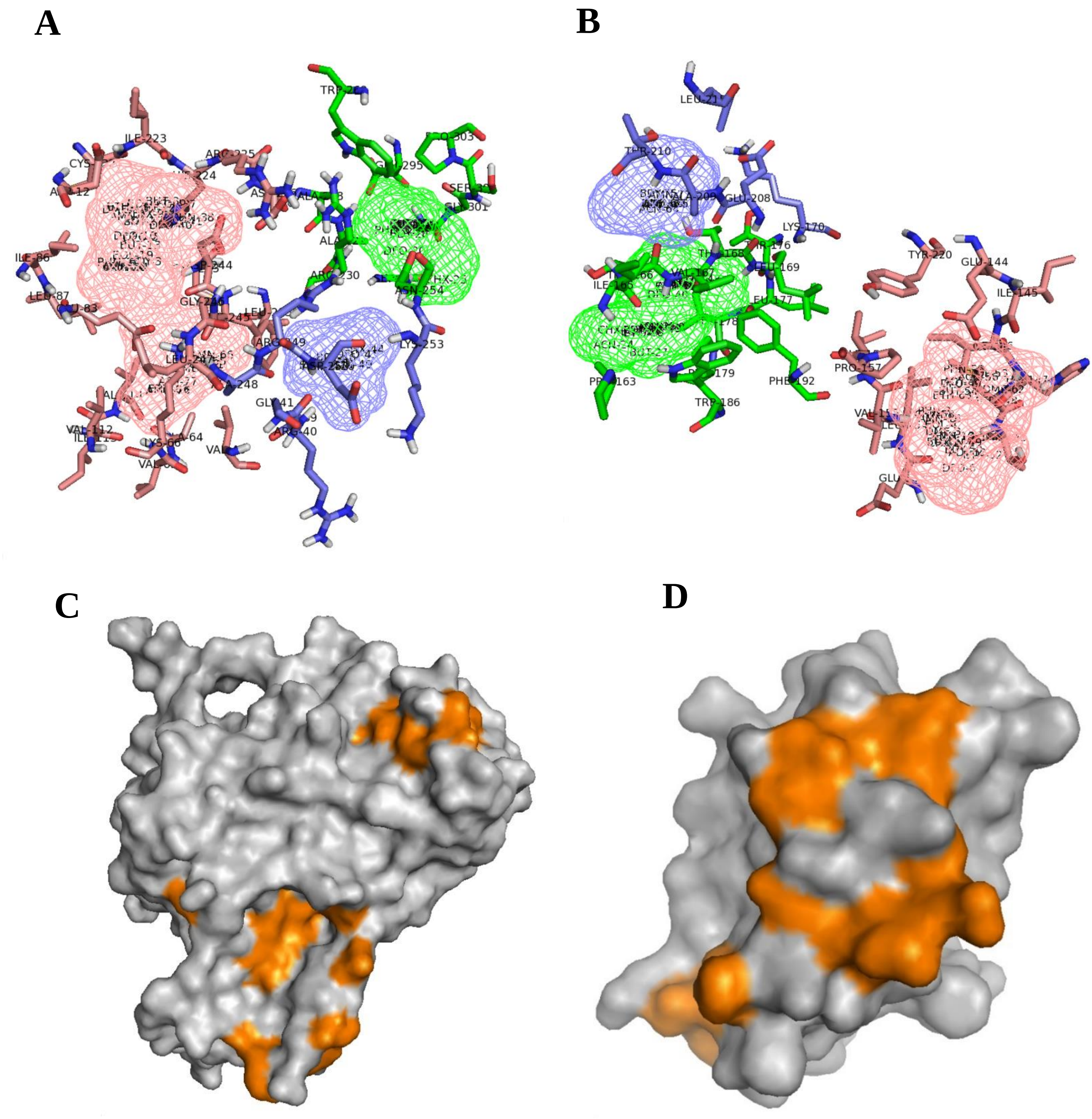

Fig. S3. Predicted ligand binding sites in VEGFR1 proteins (A) Represents the predicted binding sites in mVEGFR1 using FTsite **(B)** Represents the predicted binding sites in hVEGFR1 using FTsite. Binding pockets were represented in wire frame and sticks **(C,D)** Represents the binding pockets predicted in mouse and human VEGFR1 using the scoring function. Binding site residues were shown in orange color molecular surface.

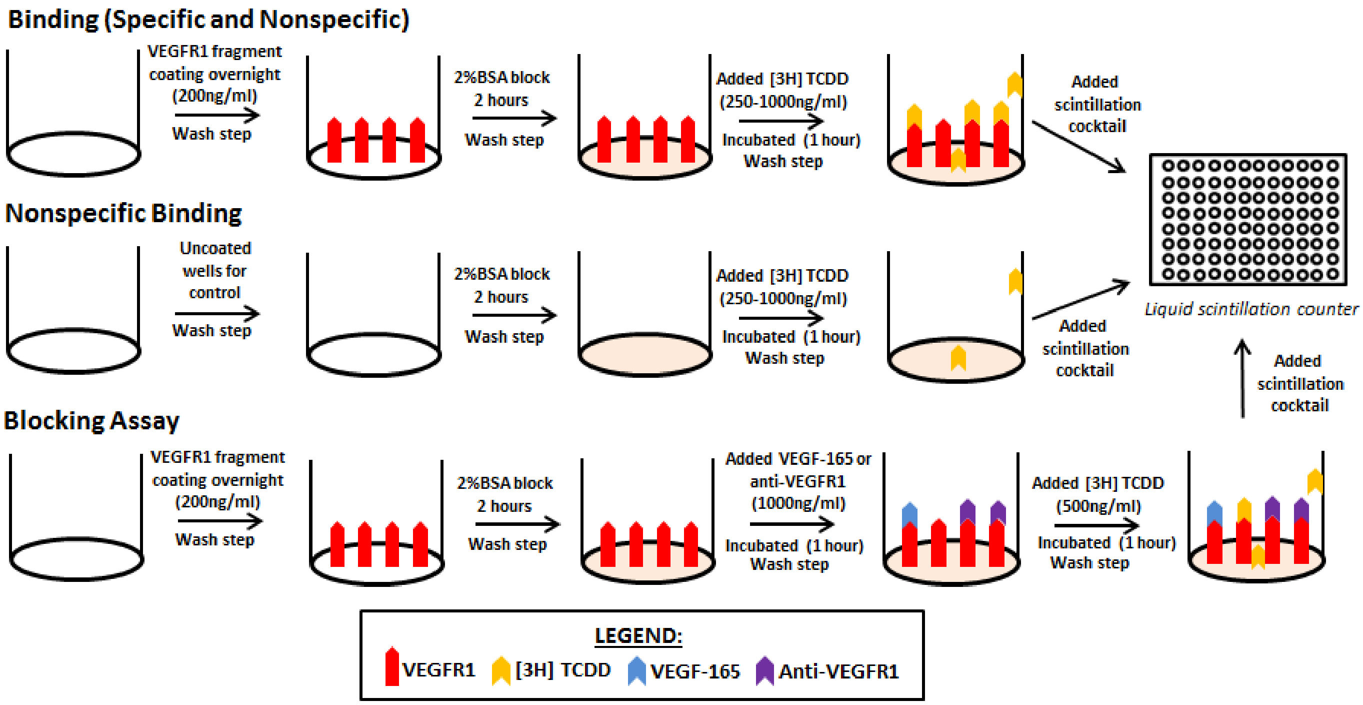

Fig. S4. Schematic representation of *in vitro* TCDD-binding and blocking assays performed in this study.

Supplementary Table S1. List of protein targets predicted against TCDD using Reverse pharmacophore approach (Pharmmapper)

a) List of TCDD protein targets predicted in *Mus musculus*

Target	PDB ID	Number of Feature	Normalized Fit Score	Z-score
Antigen-binding fragment of the anti-digoxin monoclonal antibody	1IGJ	7	0.4898	-0.127203
Fatty acid-binding protein, adipocyte	2Q9S	8	0.388	-1.06662
Peroxisomal carnitine O-octanoyltransferase	1XMC	5	0.6057	-0.353633

b) List of TCDD protein targets predicted in *Homosapiens*

Target	PDB ID	Number of Feature	Normalized Fit Score	Z-score
Transthyretin	1RLB	10	0.5121	1.68493
Retinol-binding protein 4	1RBP	8	0.6311	2.13829
retinol dehydratase	1X8L	9	0.553	1.53367
Bacteriorhodopsin	1F4Z	9	0.5392	1.55325
Acetylcholinesterase	1HBJ	9	0.5087	0.213043
Epididymal-specific lipocalin-5	1EPB	9	0.5048	1.29296
Retinol-binding protein I, cellular	1CRB	9	0.4963	1.07609
Rhodopsin	1F88	9	0.4876	0.799897
Retinol-binding protein 4	1IIU	8	0.541	1.23575
Medium-chain specific acyl-CoA dehydrogenase, mitochondrial	3MDE	6	0.7106	0.57271

Supplementary Table S2. List of protein targets predicted against TCDD using knowledge based approach (SwissProteinTarget)

a) List of TCDD protein targets predicted in *Mus musculus*

Target	Common name	Uniprot ID	Probability	Target Class
Vascular endothelial growth factor receptor 1	Flt1	P35969	1	Tyr Kinase
Aryl hydrocarbon receptor	Ahr	Q3U5D9	1	Transcription Factor
Protein Akr1b10	Akr1b10	D3Z494	0.08	Enzyme
Aldose reductase-related protein 1	Akr1b7	P21300	0.08	Enzyme
Aldose reductase	Akr1b1	P45376	0.08	Enzyme
Aldose reductase-related protein 2	Akr1b8	P45377	0.08	Enzyme
1,5-anhydro-D-fructose reductase	Akr1e2	Q9DCT1	0.08	Enzyme
Fatty acid-binding protein, intestinal	Fabp2	P55050	0.08	Cytosolic other
Beta-secretase 1	Bace1	P56818	0.07	Aspartic Protease
Beta-secretase 2	Bace2	Q9JL18	0.07	Aspartic Protease
Carbonyl reductase [NADPH] 1	Cbr1	P48758	0.04	Enzyme
Amine oxidase [flavin-containing] A	Maoa	Q64133	0.03	Enzyme
Amine oxidase [flavin-containing] B	Maob	Q8BW75	0.03	Enzyme
Arachidonate12-lipoxygenase, leukocyte-type	Alox12l	P39654	0.03	Enzyme
Arachidonate 12-lipoxygenase, 12S-type	Alox12	P39655	0.03	Enzyme

b) List of TCDD protein targets predicted in *Homosapiens*

**Target**	**Common name**	**Uniprot ID**	**Probability**	**Target Class**
Vascular endothelial growth factor receptor 1	FLT1	P17948	1	Tyr Kinase
Aryl hydrocarbon receptor	AHR	P35869	1	Transcription Factor
Vascular endothelial growth factor receptor 3	FLT4	P35916	1	Tyr Kinase
Vascular endothelial growth factor receptor 2	KDR	P35968	1	Tyr Kinase
Aldose reductase	AKR1B1	P15121	0.08	Enzyme
Aldo-keto reductase family 1 member B15	AKR1B15	C9JRZ8	0.08	Enzyme
Aldo-keto reductase family 1 member B10	AKR1B10	O60218	0.08	Enzyme
Alcohol dehydrogenase [NADP(+)]	AKR1A1	P14550	0.08	Enzyme
5-anhydro-D-fructose reductase	AKR1E2	Q96JD6	0.08	Enzyme
Fatty acid-binding protein intestinal	FABP2	P12104	0.08	Cytosolic other
Beta-secretase 1	BACE1	P56817	0.07	Aspartic Protease
Beta-secretase 2	BACE2	Q9Y5Z0	0.07	Aspartic Protease
Carbonyl reductase [NADPH] 1	CBR1	P16152	0.04	Enzyme
Carbonyl reductase [NADPH] 3	CBR3	O75828	0.04	Enzyme
Tyrosyl-DNA phosphodiesterase 1	TDP1	Q9NUW8	0.04	Enzyme

Supplementary Table S3. Calculated scores for the binding pocket residues in the mouse and human VEGFR1. The complete description of each values was provided in the Supplementary dataset 1.

Mouse Vascular endothelial growth factor receptor 1

Amino acid number	*TCa*	*Pa*	*OPc*	*EPc*	*OPc/EPc*	*Population Mean*	*Poisson Distribution*	*Poisson p-value*
TRP25	1878	1048	2	536.2790191	0.003729402	0.01411845	0.970439022	1.7984E-228
PHE133	1878	1048	2	536.2790191	0.003729402	0.01411845	0.970439022	1.7984E-228
LEU122	1878	1048	2	536.2790191	0.003729402	0.01411845	0.970439022	1.7984E-228
LEU145	1878	1048	2	536.2790191	0.003729402	0.01411845	0.970439022	1.7984E-228
LYS55	1878	1048	3	536.2790191	0.005594103	0.01411845	0.962760264	3.2148E-226
GLU26	1878	1048	2	536.2790191	0.003729402	0.01411845	0.970439022	1.7984E-228
GLU144	1878	1048	2	536.2790191	0.003729402	0.01411845	0.970439022	1.7984E-228
PHE27	1878	1048	3	536.2790191	0.005594103	0.01411845	0.962760264	3.2148E-226
LYS56	1878	1048	4	536.2790191	0.007458804	0.01411845	0.955142266	4.3101E-224
PRO160	1878	1048	2	536.2790191	0.003729402	0.01411845	0.970439022	1.7984E-228
GLY53	1878	1048	1	536.2790191	0.001864701	0.01411845	0.978179023	6.707E-231
TYR119	1878	1048	8	536.2790191	0.014917608	0.01411845	0.92526831	2.122E-216
LYS127	1878	1048	8	536.2790191	0.014917608	0.01411845	0.92526831	2.122E-216
LYS146	1878	1048	8	536.2790191	0.014917608	0.01411845	0.92526831	2.122E-216
ARG130	1878	1048	11	536.2790191	0.020511711	0.01411845	0.903477635	3.3058E-211
VAL63	1878	1048	6	536.2790191	0.011188206	0.01411845	0.940086629	4.1319E-220
ILE86	1878	1048	9	536.2790191	0.016782309	0.01411845	0.917946973	1.2644E-214
LEU87	1878	1048	9	536.2790191	0.016782309	0.01411845	0.917946973	1.2644E-214
ILE113	1878	1048	9	536.2790191	0.016782309	0.01411845	0.917946973	1.2644E-214
GLU148	1878	1048	7	536.2790191	0.013052907	0.01411845	0.93264804	3.1655E-218
MET143	1878	1048	8	536.2790191	0.014917608	0.01411845	0.92526831	2.122E-216
TYR125	1878	1048	14	536.2790191	0.026105813	0.01411845	0.882200144	2.3345E-206
VAL46	1878	1048	8	536.2790191	0.014917608	0.01411845	0.92526831	2.122E-216
VAL65	1878	1048	8	536.2790191	0.014917608	0.01411845	0.92526831	2.122E-216

VAL112	1878	1048	8	536.2790191	0.014917608	0.01411845	0.92526831	2.122E-216
VAL114	1878	1048	8	536.2790191	0.014917608	0.01411845	0.92526831	2.122E-216
MET200	1878	1048	11	536.2790191	0.020511711	0.01411845	0.903477635	3.3058E-211
GLU83	1878	1048	10	536.2790191	0.01864701	0.01411845	0.910683567	6.7808E-213
ASP131	1878	1048	8	536.2790191	0.014917608	0.01411845	0.92526831	2.122E-216
ALA140	1878	1048	5	536.2790191	0.009323505	0.01411845	0.947584547	4.6229E-222
ASN124	1878	1048	10	536.2790191	0.01864701	0.01411845	0.910683567	6.7808E-213
LYS129	1878	1048	30	536.2790191	0.055941029	0.01411845	0.776900895	3.5909E-184
SER123	1878	1048	9	536.2790191	0.016782309	0.01411845	0.917946973	1.2644E-214
SER194	1878	1048	11	536.2790191	0.020511711	0.01411845	0.903477635	3.3058E-211
SER128	1878	1048	17	536.2790191	0.031699916	0.01411845	0.861423753	8.825E-202

Human Vascular endothelial growth factor receptor 1

Amino acid number	***TCa***	***Pa***	***OPc***	***EPc***	***OPc/EPc***	***Population Mean***	***Poisson Distribution***	***Poisson p-value***
ARG133	271	558	4	202.9771812	0.019706649	6.357142857	0.001798703	4.98617E-81
PRO134	271	558	3	202.9771812	0.014779987	6.357142857	0.001782387	9.82607E-83
PHE135	271	558	4	202.9771812	0.019706649	6.357142857	0.001798703	4.98617E-81
VAL136	271	558	9	202.9771812	0.04433996	6.357142857	0.001882549	1.13619E-73
GLU137	271	558	8	202.9771812	0.039413297	6.357142857	0.001865473	5.03787E-75
SER140	271	558	2	202.9771812	0.009853324	6.357142857	0.001766219	1.45229E-84
GLU141	271	558	1	202.9771812	0.004926662	6.357142857	0.001750198	1.43099E-86
ILE142	271	558	8	202.9771812	0.039413297	6.357142857	0.001865473	5.03787E-75
PRO143	271	558	4	202.9771812	0.019706649	6.357142857	0.001798703	4.98617E-81
GLU144	271	558	10	202.9771812	0.049266622	6.357142857	0.001899782	2.30621E-72
HIS147	271	558	12	202.9771812	0.059119946	6.357142857	0.001934722	7.19812E-70
MET148	271	558	11	202.9771812	0.054193284	6.357142857	0.001917173	4.25553E-71
THR149	271	558	11	202.9771812	0.054193284	6.357142857	0.001917173	4.25553E-71
GLU150	271	558	2	202.9771812	0.009853324	6.357142857	0.001766219	1.45229E-84

Supplementary Table S4. Calculated scores for the binding pocket residues in the mouse and human VEGFR1 using Castp server. High ranked pockets were shown in bold letters. Vol_ms refers to the binding cavity volume calculated using Connolly's molecular surface.

a)

Pocket number	Vol_ms	Population Mean	Population standard deviation	Z-score
1	1.66	206.3807692	557.5699158	-0.367166096
2	14.69	206.3807692	557.5699158	-0.343796829
3	14.43	206.3807692	557.5699158	-0.344263139
4	12.56	206.3807692	557.5699158	-0.347616978
5	11.05	206.3807692	557.5699158	-0.350325159
6	16.08	206.3807692	557.5699158	-0.341303868
7	17.91	206.3807692	557.5699158	-0.338021769
8	21.91	206.3807692	557.5699158	-0.330847781
9	14.65	206.3807692	557.5699158	-0.343868569
10	24.26	206.3807692	557.5699158	-0.326633063
11	27.82	206.3807692	557.5699158	-0.320248213
12	17.68	206.3807692	557.5699158	-0.338434273
13	25.15	206.3807692	557.5699158	-0.32503685
14	35.1	206.3807692	557.5699158	-0.307191555
15	21.46	206.3807692	557.5699158	-0.331654854
16	40.64	206.3807692	557.5699158	-0.297255581
17	40.52	206.3807692	557.5699158	-0.297470801
**18**	**70.42**	**206.3807692**	**557.5699158**	**-0.243845239**
19	40.09	206.3807692	557.5699158	-0.298242004
20	35.88	206.3807692	557.5699158	-0.305792627
21	55.58	206.3807692	557.5699158	-0.270460735
**22**	**72.53**	**206.3807692**	**557.5699158**	**-0.24006096**
**23**	**95.52**	**206.3807692**	**557.5699158**	**-0.198828463**
24	574.69	206.3807692	557.5699158	0.660561519
25	1672.54	206.3807692	557.5699158	2.629552257
26	2391.08	206.3807692	557.5699158	3.918251629

b)

Pocket number	Vol_ms	Population Mean	Population standard deviation	Z-score
1	7.3	30.73636364	36.27782443	-0.646024507
2	13.3	30.73636364	36.27782443	-0.480634214
3	17	30.73636364	36.27782443	-0.378643534
4	14.6	30.73636364	36.27782443	-0.444799651
5	12	30.73636364	36.27782443	-0.516468778
6	13.7	30.73636364	36.27782443	-0.469608195
**7**	**21.4**	**30.73636364**	**36.27782443**	**-0.257357319**
**8**	**21.8**	**30.73636364**	**36.27782443**	**-0.2463313**
**9**	**20.9**	**30.73636364**	**36.27782443**	**-0.271139844**
10	67.3	30.73636364	36.27782443	1.00787842
11	128.8	30.73636364	36.27782443	2.70312892

Supplementary Table S5. Calculated scores for the binding pocket residues in the mouse and human VEGFR1 using Active site server. High ranked pockets were shown in bold letters.

a)

Pocket number	Vol_ms	Population Mean	Population standard deviation	Z-score
1	846	290.2222222	263.7611566	2.107125192
2	966	290.2222222	263.7611566	2.562082251
3	427	290.2222222	263.7611566	0.518566796
4	497	290.2222222	263.7611566	0.783958413
**5**	**238**	**290.2222222**	**263.7611566**	**-0.197990572**
6	469	290.2222222	263.7611566	0.677801766
**7**	**358**	**290.2222222**	**263.7611566**	**0.256966487**
**8**	**209**	**290.2222222**	**263.7611566**	**-0.307938528**
9	193	290.2222222	263.7611566	-0.368599469
10	120	290.2222222	263.7611566	-0.645365013
11	143	290.2222222	263.7611566	-0.55816491
12	178	290.2222222	263.7611566	-0.425469101
13	195	290.2222222	263.7611566	-0.361016851
14	67	290.2222222	263.7611566	-0.84630438
15	75	290.2222222	263.7611566	-0.81597391
16	100	290.2222222	263.7611566	-0.721191189
17	73	290.2222222	263.7611566	-0.823556527
18	70	290.2222222	263.7611566	-0.834930454

b)

Pocket number	Vol_ms	Population Mean	Population standard deviation	Z-score
1	102	257	21.73323108	-7.131935396
2	94	257	21.73323108	-7.500035287
3	61	257	21.73323108	-9.018447339

Supplementary data 1. Description of the scoring parameters used to predict the amino acid residues lining the pocket for mouse and human VEGFR1

We computed the expected pocket conservation, EP_c to predict the binding pocket residues for mVEGFR1 and hVEGFR1:

$$EP_c = TC_a * P_a / T_a \qquad (1)$$

where EP_c = Expected pocket conservation

TC_a = Total number of atoms belonging to the conserved amino acids

P_a = Number of atoms lining pocket

T_a = Total number of atoms of the protein

From the equation Poisson distribution and Poisson probability was calculated using the following equations

$$\text{Poisson distribution} = \lambda^{OPc / EPc} e^{-\lambda} / (OPc / EPc)! \qquad (2)$$

where λ is the population mean and e is equal to 2.71828

$$\text{Poisson probability } p \text{ value} = e^{-EPc} EPc^{OPc} / OP_c! \qquad (3)$$

where OP_c = The observed number of atoms lining the pocket, and belonging to amino acids conserved in alignment

We identified the pockets enriched in conserved amino acids with a greater OP_C/EP_C value at a level of statistical significance, Poisson *p*-value, lower than 0.05. The residues with greater OP_c / EP_c and lesser poisson p-value ($p < 0.05$) were considered as the pocket residues.
